# Systemic Oidiomycosis, with Manifestations in Central Nervous System

**Published:** 1917-07

**Authors:** 


### ABSTRACTS

**Systemic Oidiomycosis, with Manifestations in Central Nervous System.** Frederic J. Farnell and Samuel Starr of Providence, Boston M. & S. Jour., May 31, report a case beginning with a felon the terminal phalanx showing a moth-eaten appearance by X-ray. The pulmonary lesions suggested tuberculosis but no tubercle bacilli were found. The rather vaguely described symptoms suggested meningitis. Wassermann negative. An injection of salvarsan increased the symptoms. Pus was found in the right pleura and over the shin. Oidium and streptococcus viridans were isolated. Iodids gave relief. Stober believes the extra-corporeal habitat of the oidium to be rotten wood and leaves. Reference is made to a study by Stoddard and Cutler, monograph issued by Rockefeller Institute, of the relations of coccidioid granuloma, blastomycosis (oidiomycosis) and torula infection.

